# Isorhamnetin Attenuated the Release of Interleukin-6 from *β*-Amyloid-Activated Microglia and Mitigated Interleukin-6-Mediated Neurotoxicity

**DOI:** 10.1155/2022/3652402

**Published:** 2022-09-15

**Authors:** Pei-Cih Wei, Guey-Jen Lee-Chen, Chiung-Mei Chen, Ying Chen, Yen-Shi Lo, Kuo-Hsuan Chang

**Affiliations:** ^1^Department of Neurology, Chang Gung Memorial Hospital-Linkou Medical Center, Chang Gung University School of Medicine, Taoyuan, Taiwan; ^2^Department of Life Science, National Taiwan Normal University, Taipei, Taiwan

## Abstract

Alzheimer's disease (AD), characterized by the abnormal accumulation of *β*-amyloid (A*β*), is the most prevalent type of dementia, and it is associated with progressive cognitive decline and memory loss. A*β* accumulation activates microglia, which secrete proinflammatory factors associated with A*β* clearance impairment and cause neurotoxicity, generating a vicious cycle among A*β* accumulation, activated microglia, and proinflammatory factors. Blocking this cycle can be a therapeutic strategy for AD. Using A*β*-activated HMC3 microglial cells, we observed that isorhamnetin, a main constituent of *Oenanthe javanica*, reduced the A*β*-triggered secretion of interleukin- (IL-) 6 and downregulated the expression levels of the microglial activation markers ionized calcium binding adaptor molecule 1 (IBA1) and CD11b and the inflammatory marker nuclear factor-*κ*B (NF-*κ*B). Treatment of the SH-SY5Y-derived neuronal cells with the A*β*-activated HMC3-conditioned medium (HMC3-conditioned medium) or IL-6 increased reactive oxygen species production, upregulated cleaved caspase 3 expression, and reduced neurite outgrowth, whereas treatment with isorhamnetin counteracted these neurodegenerative presentations. In the SH-SY5Y-derived neuronal cells, IL-6 upregulated the phosphorylation of tyrosine kinase 2 (TYK2) and signal transducer and activator of transcription 1 (STAT1), whereas isorhamnetin normalized this abnormal phosphorylation. Overexpression of TYK2 attenuated the neuroprotective effect of isorhamnetin on IL-6-induced neurotoxicity. Our findings demonstrate that isorhamnetin exerts its neuroprotective effect by mediating the neuroinflammatory IL-6/TYK2 signaling pathway, suggesting its potential for treating AD.

## 1. Introduction

Alzheimer's disease (AD), the most common cause of dementia in older people, is an irreversible and a progressive neurodegenerative disease that slowly impairs cognitive function, memory and thinking, and eventually the ability to perform the simplest daily tasks [1]. With the rapid increase in the aging population in developing and developed countries, AD is becoming a major health problem globally [2]. However, the lack of effective treatment for preventing disease progression necessitates the development of new agents that can halt the pathogenesis of AD. Pathologically, AD is characterized by the presence of senile plaques in the brain [1]. Senile plaques are composed of the *β*-amyloid (A*β*) peptide, a fragment of the amyloid peptide precursor protein (APP) [3]. A*β* peptides aggregate to form oligomers and other high-order polymerized structures that cause neuronal death through various mechanisms including neuroinflammation, oxidative stress, excitotoxicity, energy depletion, and apoptosis [4].

Neuroinflammation is a prominent feature in AD pathology and a potential target for the treatment and prevention of AD [5]. Neuroinflammation links A*β* peptide deposition to neuronal death by activating microglia through Toll-like receptor 2 [6]. Activated microglia secrete proinflammatory factors including interleukin- (IL-) 1*β* [7], IL-6 [8], and tumor necrosis factor- (TNF-) *α* [7]. The expression levels of IL-1*β*, IL-6, and TNF-*α* have been reported to be upregulated in the brains of AD animal models [7, 9] and patients with AD [1]. In addition, an increased risk of AD is associated with the genetic variants of IL-1*β* [10], IL-6 [11], and TNF-*α* [12]. Abnormal production of proinflammatory factors causes neurotoxicity and impairs A*β* peptide clearance, forming a vicious cycle among A*β* peptide accumulation, activated microglia, and proinflammatory factors. Blocking this cycle can be a vital strategy for halting neurodegeneration in AD.

Isorhamnetin, a small-molecular compound with an aromatic heterocyclic structure, is the main constituent of the *Oenanthe javanica* extract. Isorhamnetin exerts an anti-inflammatory effect by modulating I*κ*B kinase (IKK)*α*, IKK*β*, and nuclear factor- (NF-) *κ*B [13]. In a rat model, isorhamnetin demonstrated neuroprotection against A*β* peptides [14]. This study investigated the effects of isorhamnetin on microglial activation and neuroinflammation in the cell models of AD. Isorhamnetin inhibited A*β* peptide-mediated microglial activation and exerted a neuroprotective effect on IL-6-mediated neurotoxicity. These findings support the potential of isorhamnetin for use in AD treatment.

## 2. Materials and Methods

### 2.1. Maintenance and Neural Induction of SH-SY5Y Cells

SH-SY5Y cells (American Type Culture Collection (ATCC) CRL-2266) were maintained in Dulbecco's modified Eagle medium/nutrient mixture F-12 (DMEM/F12) containing 10% fetal bovine serum (FBS) at 37°C under 5% CO_2_. Subsequently, for neuronal differentiation, 20 *μ*M retinoic acid (RA) was added to the culture medium and the cells were incubated for 5 days. The differentiated cells were treated with isorhamnetin (10 *μ*M, Merck), TYK2 inhibitor (1 *μ*M, BMS-986165, AdooQ), or IL-6-neutralized antibody (IL-6 IgG, 5 ng/mL, InvivoGen) for 2 days, respectively.

### 2.2. HMC3 Cell Culture and Drug Treatment

HMC3 cells (human microglial cells; ATCC CRL-3304) were routinely cultured in DMEM/F12 containing 10% FBS at 37°C under 5% CO2 and 95% relative humidity. The HMC3 cells were activated by the addition of A*β* oligomers (200 nM) for 24 or 48 hours and then treated with isorhamnetin (10 *μ*M) or NF-*κ*B inhibitor EVP4593 (1 *μ*M, Merck) for 48 hours.

### 2.3. Cell Viability Assessment Using Lactate Dehydrogenase and 2,5-Diphenyl-2H-Tetrazolium Bromide Assays

According to the manufacturer's instructions (Roche), the cells were incubated with 100 *μ*L of lactate dehydrogenase (LDH) reagent at room temperature for 20 min. The absorbance of the samples was measured at 490 nm by using a spectrophotometer. For the 2,5-diphenyl-2H-tetrazolium bromide (MTT) assay, the cells were cultivated with 20 *μ*L of MTT (5 mg/mL in phosphate-buffered saline (PBS)) and incubated at 37°C for 2 hours. Subsequently, the formation of purple formazan crystals was determined by measuring absorbance at 570 nm by using a microplate spectrophotometer.

### 2.4. Preparation of A*β* Oligomers [15]

A*β*42 peptides were dissolved in hexafluoro-2-propanol (HFIP) and incubated at room temperature for at least 1 hour. To obtain the peptide film, HFIP was removed through evaporation and the resulting peptides were stored at −20°C or −80°C. The resulting film was adjusted to a concentration of 5 mM by using dimethyl sulfoxide and then diluted to achieve appropriate concentrations for experiments.

### 2.5. Western Blotting

Total proteins were lysed using RIPA buffer containing a protease inhibitor cocktail (Sigma) and a phosphatase inhibitor (Sigma). Thereafter, 60 *μ*g of proteins was separated through 12% sodium dodecyl sulfate-polyacrylamide gel electrophoresis and transferred onto polyvinylidene fluoride membranes. The blocked membrane was hybridized using the following antibodies: glyceraldehyde 3-phosphate dehydrogenase (GAPDH) (1 : 10,000 dilution, Proteintech), NF-*κ*B (1 : 1000 dilution, Cell Signaling), p-NF-*κ*B/p65 (1 : 1000 dilution, Cell Signaling), ionized calcium-binding adaptor molecule 1 (IBA1) (1 : 1000 dilution, WAKO), cleaved caspase 3 (dilution 1 : 1000, Cell Signaling), CD11b (1 : 1000 dilution, WAKO), CD68 (1 : 1000 dilution, Boster), p-IKK*α*/p-IKK-*ꞵ* (1 : 1000 dilution, Cell Signaling), IKK*α* (1 : 1000 dilution, Cell Signaling), IKK*ꞵ* (1 : 1000 dilution, Cell Signaling), Janus kinase 2 (JAK2) (1 : 1000 dilution, Cell Signaling), p-JAK2 (1 : 1000 dilution, Cell Signaling), tyrosine kinase 2 (TYK2) (1 : 1000 dilution, Cell Signaling), p-TYK2 (1 : 1000 dilution, Cell Signaling), signal transducer and activator of transcription 3 (STAT3) (1 : 1000 dilution, Santa Cruz), p-STAT3 (1 : 1000 dilution, Santa Cruz), signal transducer and activator of transcription 1 (STAT1) (1 : 1000 dilution, Cell Signaling), and p-STAT1 (1 : 1000 dilution, Cell Signaling). The protein expression was detected using horseradish peroxidase-labeled goat anti-mouse or goat anti-rabbit IgG antibody and a chemiluminescent substrate.

### 2.6. Transfection with Plasmid DNA

The SH-SY5Y cells were transfected with the TYK2 expression vector (Sino Biological) by using the X-treme transfection reagent (Roche) according to the manufacturer's instructions. Subsequently, the expression of TYK2 was confirmed through Western blotting after 48 hours of transfection.

### 2.7. Reactive Oxidative Species Analysis

The cells were seeded in six-well plates (10^5^ cells/well) and differentiated into neuronal cells through treatment with retinoic acid (RA) (20 *μ*M) for 5 days. Subsequently, the neuronal cells were treated with isorhamnetin or IL-6 for 48 hours. For reactive oxygen species (ROS) analysis, the fluorogenic CellROX deep red reagent (Molecular Probes) was added to the live cells, followed by incubation at 37°C for 6 hours. Thereafter, the cells were washed with PBS and red fluorescence (indicating the presence of ROS) was examined using a Leica TCS confocal microscope at the excitation and emission wavelengths of 644 and 665 nm, respectively.

### 2.8. Neurite Outgrowth Evaluation Using Immunofluorescence Staining

Neurons were cultured on a coverslip and washed with PBS. Subsequently, they were fixed with 4% paraformaldehyde for 20 min at room temperature. After the residual fixation buffer was removed using PBS, the cells were blocked in PBS containing 5% BSA for 20 min at room temperature and then hybridized with the primary antibody tubulin class III (TUBB3) (1 : 5000 dilution, BioLegend) overnight at 4°C. After being washed with PBS, the cells were incubated with an Alexa 488-conjugated secondary antibody in the dark for 1 hour. For cell counting, the cells were stained with 4′,6-diamidino-2-phenylindole (1 : 1000 dilution). Subsequently, the mounted coverslips were examined using a Leica TCS confocal microscope. Neurite outgrowth on the SH-SY5Y-derived neurons (>200 cells) was analyzed using MetaMorph software (Molecular Devices).

### 2.9. Preparation of HMC3-Conditional Medium

The HMC3 cells were treated by A*ꞵ* oligomers (200 nM) for 48 hours, and the medium was replaced with fresh culture medium for 24 hours. The supernatant free from A*ꞵ* oligomers was collected for further experiments.

### 2.10. ELISA

The conditional medium (2 mL) was collected from a 3.5 cm dish seeded with 10^5^ HMC3 cells/well after treatment with A*ꞵ* oligomers (200 nM) for 24 or 48 hours, followed by isorhamnetin (10 *μ*M) for 48 hours. The collected medium was centrifuged at 1500 rpm for 5 min at room temperature. We used 100 *μ*L of the supernatant for the assessment. The concentrations of the proinflammatory cytokines/factors TNF-*α*, IL-1*β*, and IL-6 were analyzed using commercially available ELISA kits according to the manufacturer's protocol (IL-1*β*: Thermo Fisher; IL-6: R&D; TNF-*α*: R&D).

### 2.11. Statistical Analyses

All statistical analyses were performed using Student's *t*-test or one-way analysis of variance (ANOVA) with Bonferroni's post hoc test by using SPSS 18.0 (SPSS Inc., Chicago, IL, USA).

## 3. Results

### 3.1. Activation of Human Microglia by the Synthetic A*β* Oligomer

**Figure 1:**
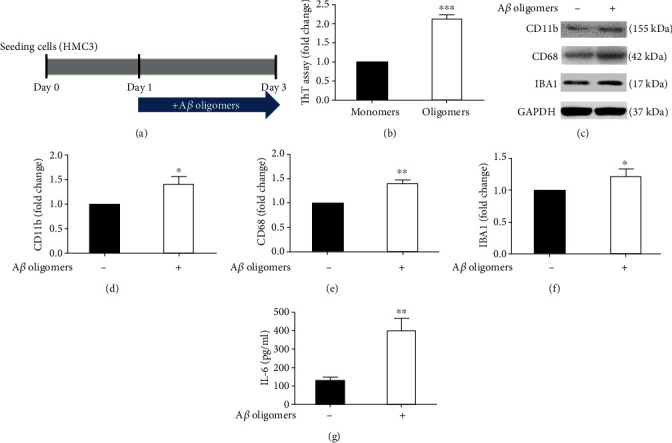
Activation of HMC3 microglia by A*β* oligomers. (a) Experimental flowchart. HMC3 cells were treated with A*β* oligomers (200 nM) for 48 hours. (b) The thioflavin T (ThT) assay demonstrated the misfolding of A*β* by oligomerization. (c–f) Treatment with A*β* oligomers upregulated the expression of the microglia activation markers CD11b, CD68, and IBA1. (g) Treatment with A*β* oligomers increased the IL-6 concentration in the culture medium. Data were analyzed using Student's *t*-test (*n* = 3, independent assays; ^∗^*P* < 0.05, ^∗∗^*P* < 0.01, ^∗∗∗^*P* < 0.001, means ± SEM).

Studies have revealed that A*β* oligomers activate microglia to secrete proinflammatory factors [7, 8]. Therefore, we treated the human microglial HMC3 cells with A*β* oligomers [15, 16] (Figure 1(a)). A*β* oligomers demonstrated higher fluorescent signals in the thioflavin T assay than did monomeric A*β* (fold change: 2.13, *P* < 0.001, Figure 1(b)). Treatment of the HMC3 cells with A*β* oligomers for 48 hours increased the expression levels of the microglial activation markers CD11b [17] (fold change: 1.40, *P* < 0.05, Figures 1(c) and 1(d)), CD68 [17] (fold change: 1.41, *P* < 0.01, Figures 1(c) and 1(e)), and IBA1 [17] (fold change: 1.21, *P* < 0.05, Figures 1(c) and 1(f)) and the secretion of IL-6 (treatment vs no treatment: 399.60 vs 129.85 pg/mL, *P* < 0.01, Figure 1(g)). By contrast, the secretions of IL-1*β* (treatment vs no treatment: 0.42 pg/mL vs 0.34 pg/mL, Supplementary Figure 1A) and TNF-*α* (treatment vs no treatment: 6.73 pg/mL vs 11.21 pg/mL, Supplementary Figure 1A) were not altered by treatment with A*β* oligomers. Twenty-four-hour treatment with A*β* oligomers to HMC3 cells demonstrated consistent results of cytokine releases (Supplementary Figure 1A). These results demonstrated that A*β* oligomers can activate microglia and increase IL-6 secretion from microglia. Given 48 hours treatment with A*β* oligomers to HMC3 cells, it generated relatively pronounced IL-6 secretion compared with 24 hours treatment. Therefore, we chose this condition for further experiments.

### 3.2. Isorhamnetin Reduced A*β*-Mediated Microglial Activation

**Figure 2:**
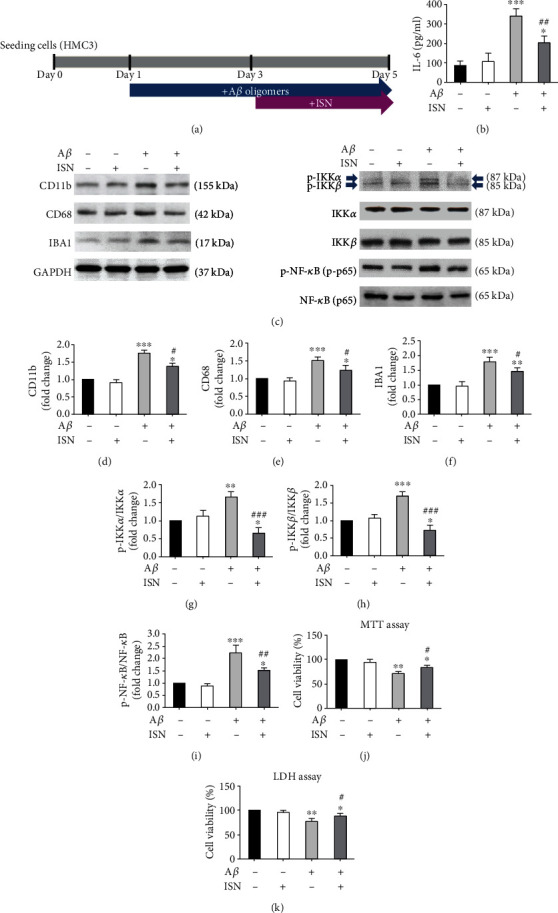
Isorhamnetin reduced the IL-6 concentration and NF-*κ*B/p65 phosphorylation in A*β*-activated microglial cells. (a) Experimental flowchart. (b) A*β* (200 nM) increased the secretion of IL-6 on HMC cells, whereas cotreatment with isorhamnetin (10 *μ*M) reduced the secretion of IL-6. (c–i) HMC3 cells with the upregulated expression of the microglial activation markers CD11b, CD68, and IBA1 increased the phosphorylation of IKK*α*, IKK*β*, and NF-*κ*B. Isorhamnetin cotreatment reduced the expression of CD11b, CD68, and IBA1 and normalized the phosphorylation of IKK*α*, IKK*β*, and NF-*κ*B. (j, k) A*β* reduced cell viability, as observed in MTT and LDH assays, whereas isorhamnetin improved cell viability. Data were analyzed using one-way ANOVA with Bonferroni's post hoc test (^∗^*P* < 0.05, ^∗∗^*P* < 0.01, and ^∗∗∗^*P* < 0.001 (control vs A*β*); ^#^*P* < 0.05, ^##^*P* < 0.01, and ^###^*P* < 0.001 (A*β* vs A*β*/ISN), *n* = 3; means ± SEM). A*β*: A*β* oligomers; ISN: isorhamnetin.

Evidence suggests that isorhamnetin has anti-inflammatory properties [18]. We examined whether isorhamnetin exerts an anti-inflammatory effect on microglia (Figure 2(a)). In the time-response experiments, treatment with isorhamnetin for 2 days demonstrated protective effects on A*β* toxicity in HMC3 cells (Supplementary Figure 1B, C). Treatment with A*β* oligomers increased the secretion of IL-6 (366.08 vs 86.92 pg/mL, *P* < 0.001 compared with no treatment, Figure 2(b)), whereas isorhamnetin significantly attenuated the increase in the IL-6 concentration (213.88 pg/mL, *P* < 0.01 compared with A*β* treatment only, Figure 2(b)) in activated HMC3. Meanwhile, A*β* oligomers also upregulated the microglial activation markers CD11b (fold change: 1.75, *P* < 0.001 compared with no treatment, Figures 2(c) and 2(d)), CD68 (fold change: 1.51, *P* < 0.001 compared with no treatment, Figures 2(c) and 2(e)), and IBA1 (fold change: 1.79, *P* < 0.001 compared with no treatment, Figures 2(c) and 2(f)) and impaired HMC3 viability, as observed in MTT (71.71%, *P* < 0.01 compared with no treatment, Figure 2(j)) and LDH assays (76.68%, *P* < 0.01 compared with no treatment, Figure 2(k)). Furthermore, isorhamnetin rescued the impairment of cell viability by A*β* oligomers (MTT: 83.28%, *P* < 0.05 compared with A*β* oligomers; LDH: 87.98%, *P* < 0.05 compared with A*β* oligomers, Figures 2(j) and 2(k)) and normalized the expression levels of CD11b (fold change: 1.37, *P* < 0.05 compared with A*β* treatment only, Figures 2(c) and 2(d)), CD68 (fold change: 1.25, *P* < 0.05 compared with A*β* treatment only, Figures 2(c) and 2(e)), and IBA1 (fold change: 1.46, *P* < 0.05 compared with A*β* treatment only, Figures 2(c) and 2(f)). In addition, treatment with A*β* oligomers increased the phosphorylation of IKK*α* (fold change: 1.61, *P* < 0.01, Figures 2(c) and 2(g)), IKK*β* (fold change: 1.70, *P* < 0.001, Figures 2(c) and 2(h)), and NF-*κ*B (fold change: 2.22, *P* < 0.001, Figures 2(c) and 2(i)), whereas isorhamnetin normalized this abnormal phosphorylation (p-IKK*α*: fold change: 0.66, *P* < 0.001 compared with A*β* treatment only, Figures 2(c) and 2(g); p-IKK*β*: fold change: 0.73, *P* < 0.001 compared with A*β* treatment only, Figures 2(c) and 2(h); and p-NF-*κ*B: fold change: 1.51, *P* < 0.01 compared with A*β* treatment only, Figures 2(c) and 2(i)). Treatment with the NF-*κ*B inhibitor (EVP4593) to A*β*-activated HMC3 cells reduced the expression of CD11b (A*β* vs A*β*/EVP4593: fold change: 1.98 vs 1.29, *P* < 0.05, Supplementary Figure 2A, C), CD68 (A*β* vs A*β*/EVP4593: fold change: 1.71 vs 1.22, *P* < 0.01, Supplementary Figure 2A, D), and IBA1 (A*β* vs A*β*/EVP4593: fold change: 2.16 vs 1.42, *P* < 0.05, Supplementary Figure 2A, E), as well as increased the secretion of IL-6 (A*β* vs A*β*/EVP4593: 389.67 pg/mL vs 256.54 pg/mL, *P* < 0.05, Supplementary Figure 2F). Taken together, these results indicated that isorhamnetin reduced A*β*-mediated activation and IL-6 secretion in microglia.

### 3.3. Isorhamnetin Had a Neuroprotective Effect on Neurons Exposed to HMC3-Activated Conditional Medium

**Figure 3:**
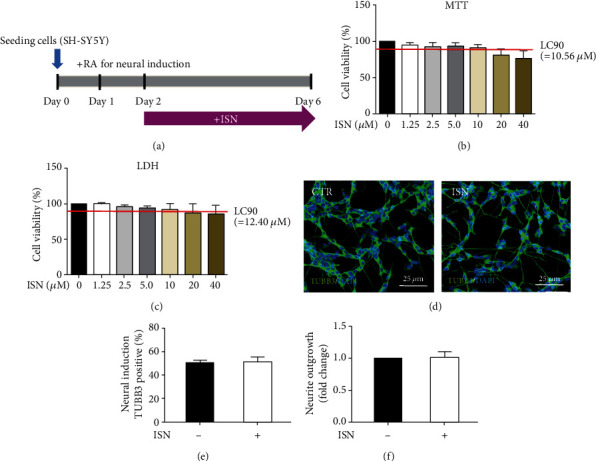
Neurotoxicity of isorhamnetin. (a) Experimental flowchart. SH-SY5Y cells were treated with isorhamnetin for 4 days. (b, c) LC_90_ determined by performing MTT and LDH assays. (d–f) Comparison of neurite outgrowth and the neuronal marker (TUBB3) between neurons treated without and with isorhamnetin (10 *μ*M). Quantification of TUBB3-positive cells and neurite outgrowth through MetaMorph software. Data were analyzed using Student's *t*-test (independent assays, *n* = 3; means ± SEM). Scale bar, 25 *μ*m. ISN: isorhamnetin.

The SH-SY5Y cells were differentiated into neuronal cells through the addition of retinoic acid (RA) [19] (Figure 3(a)). The findings of MTT and LDH assays indicated the low cytotoxicity of isorhamnetin in the SH-SY5Y cells (LC_90_: MTT: 10.56 *μ*M, Figure 3(b); LDH: 12.40 *μ*M, Figure 3(c)). Compared with no treatment, treatment with isorhamnetin resulted in a comparable number of TUBB3^+^ neurons (TUBB3^+^ neurons: isorhamnetin vs no treatment: 51.22% vs 50.38%, Figures 3(d) and 3(e)) and well-developed neurite outgrowth (neurite outgrowth, fold change: 1.02, Figures 3(d) and 3(f)). These results demonstrated that isorhamnetin resulted in low neurotoxicity and did not affect neuronal differentiation.

**Figure 4:**
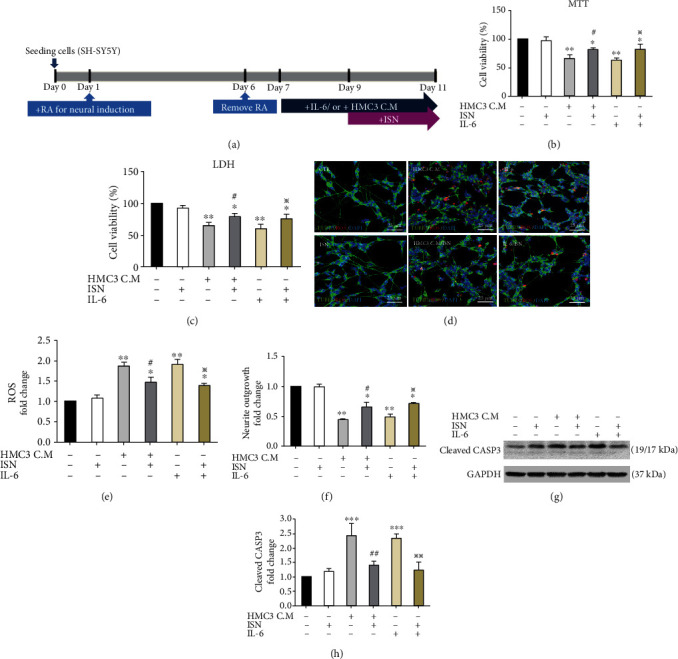
Isorhamnetin reduced ROS production, improved neurite outgrowth, and reduced apoptosis in the neurons treated with the HMC3-conditioned medium or IL-6. (a) Experimental design of neuroinflammation induced by treatment with the conditioned medium from A*β*-treated HMC3 cells or IL-6 (350 pg/mL). (b, c) Isorhamnetin (10 *μ*M) maintained the viability of SH-SY5Y cells treated with the HMC3-conditioned medium or IL-6. (d–f) Isorhamnetin reduced the ROS level and attenuated neurite outgrowth impaired by treatment with the HMC3-conditioned medium or IL-6. Images were examined using MetaMorph software. Scale bar, 25 *μ*m. (g, h) Western blotting indicated that neurons treated with the HMC3-conditioned medium or IL-6 increased the expression of cleaved caspase 3, whereas cotreatment with isorhamnetin reduced the expression of cleaved caspase 3. Data were analyzed using one-way ANOVA with Bonferroni's post hoc test (^∗^*P* < 0.05, ^∗∗^*P* < 0.01, and ^∗∗∗^*P* < 0.001 (control vs HMC3 C.M; control vs IL-6); ^#^*P* < 0.05, ^##^*P* < 0.01 (HMC3 C.M vs HMC3 C.M/ISN); ^※^*P* < 0.05, ^※※^*P* < 0.01 (IL-6 vs IL-6/ISN), *n* = 3; means ± SEM). HMC3 C.M: HMC3-conditional medium; ISN: isorhamnetin.

To investigate the anti-neuroinflammatory and neuroprotective effects of isorhamnetin, we exposed the SH-SY5Y-derived neuronal cells to the HMC3-conditioned medium and then treated them with isorhamnetin (Figure 4(a)). Given that treatment with A*β* oligomers significantly increased the secretion of IL-6 from the HMC3 cells, we treated the SH-SY5Y-derived neuronal cells with IL-6 at a concentration equal to that present in the HMC3-conditioned medium (350 pg/mL; Figure 2(b)). The results of MTT and LDH assays revealed that treatment with the HMC3-conditioned medium and IL-6 reduced cell viability (conditioned medium, MTT: 65.82%, *P* < 0.01, Figure 4(b); LDH: 62.17%, *P* < 0.01, Figure 4(c)), whereas isorhamnetin attenuated the impairment of cell viability (MTT: 81.39%, *P* < 0.05 compared with the HMC3-conditioned medium only, Figure 4(b); LDH: 79.53%, *P* < 0.05 compared with the HMC3-conditioned medium only, Figure 4(c)). Similarly, treatment with IL-6 impaired cell viability (MTT: 62.91%, *P* < 0.01, Figure 4(b); LDH: 60.34%, *P* < 0.01, Figure 4(c)), which was rescued by isorhamnetin treatment (MTT: 82.20%, *P* < 0.05 compared with IL-6 only, Figure 4(b); LDH: 76.31%, *P* < 0.05 compared with IL-6 only, Figure 4(c)).

Evidence suggests that neuroinflammation contributes to oxidative damage to neurons in AD [20]. Therefore, we assessed the production of ROS and neurite outgrowth. Treatment with the HMC3-conditioned medium increased ROS production (fold change: 1.87, *P* < 0.01 compared with control, Figures 4(d) and 4(e)) and reduced neurite outgrowth (fold change:0.43, *P* < 0.01 compared with control, Figures 4(d) and 4(f)). Isorhamnetin attenuated the increased ROS production (fold change: 1.48, *P* < 0.05 compared with the HMC3-conditional medium only, Figures 4(d) and 4(e)) and impaired neurite outgrowth (fold change: 0.58, *P* < 0.05 compared with the HMC3-conditional medium only, Figures 4(d) and 4(f)). Furthermore, ROS production (fold change: 1.91, *P* < 0.01 compared with control, Figures 4(d) and 4(e)) and neurite outgrowth impairment (fold change: 0.52, *P* < 0.01 compared with control, Figures 4(d) and 4(f)) were enhanced by IL-6 treatment, whereas isorhamnetin attenuated the increased ROS production (fold change: 1.39, *P* < 0.05 compared with IL-6 only, Figures 4(d) and 4(e)) and impaired neurite growth (fold change: 0.66, *P* < 0.05 compared with IL-6 only, Figures 4(e) and 4(f)). The expression level of cleaved caspase 3 was increased following treatment with the HMC3-conditioned medium (fold change: 2.43, *P* < 0.001 compared with control, Figures 4(g) and 4(h)) or IL-6 (fold change: 2.30, *P* < 0.001 compared with control, Figures 4(g) and 4(h)). Isorhamnetin attenuated the increase in the expression level of cleaved caspase 3 caused by treatment with the HMC3-conditioned medium (fold change: 1.40, *P* < 0.01 compared with the conditioned medium only, Figures 4(g) and 4(h)) or IL-6 (fold change:1.24, *P* < 0.01 compared with IL-6 only, Figures 4(g) and 4(h)).

To further confirm the role of IL-6 as the main toxic cytokine to neurons, we treated SH-SY5Y-derived neuronal cells with IL-6 IgG and HMC3-conditioned medium concomitantly. IL-6 IgG reduced the levels of cleaved-caspase 3 (HMC3-conditioned medium vs HMC3-conditioned medium/IL-6 IgG: fold change: 2.91 vs 1.88, *P* < 0.05, Supplementary Figure 3A-B) and ROS (HMC3-conditioned medium vs HMC3-conditioned medium/IL-6 IgG: fold change: 4.24 vs 2.55, *P* < 0.05, Supplementary Figure 3C-D) compared with those exposed to HMC3-conditioned medium only. Moreover, treatment with IL-6 IgG improve the neurite outgrowth (HMC3-conditioned medium vs HMC3-conditioned medium/IL-6 IgG: fold change: 0.50 vs 0.71, *P* < 0.05, Supplementary Figure 3C, E). Taken together, these results indicated that IL-6 is the main neurotoxic constituent in HMC3-conditioned medium. Isorhamnetin demonstrated a neuroprotective effect against this neuroinflammation-mediated neurotoxicity.

### 3.4. Isorhamnetin Exerted a Neuroprotective Effect by Modulating the TYK2/STAT1 Pathway

**Figure 5:**
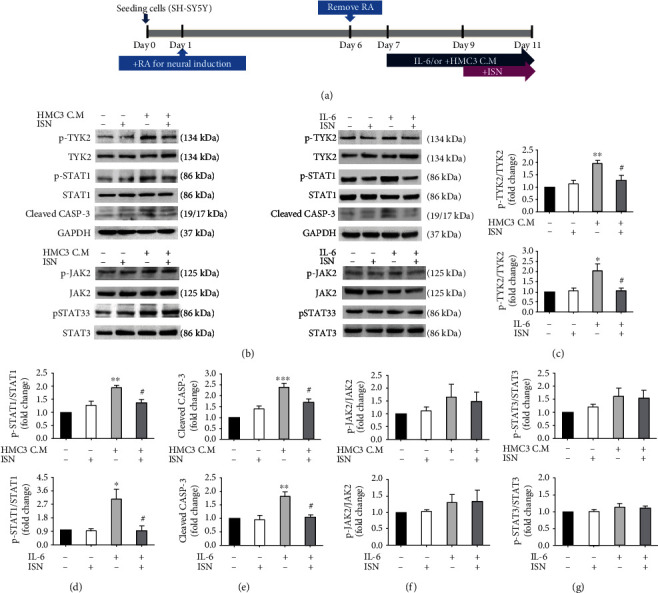
Isorhamnetin exerted neuroprotective effects by modulating the TYK2/STAT1 pathway. (a) Experimental design scheme. (b–g) Western blotting revealed the modulation of TYK2/STAT1 signaling and cleaved caspase 3 in the neurons treated with IL-6 (350 pg/mL) and/or isorhamnetin (10 *μ*M) or HMC3-conditioned medium and/or isorhamnetin. Isorhamnetin impeded the activities of the apoptotic and TYK2/STAT1 pathway. Data were analyzed using one-way ANOVA with Bonferroni's post hoc test (^∗^*P* < 0.05, ^∗∗^*P* < 0.01, and ^∗∗∗^*P* < 0.001 (control vs IL-6; control vs IL-6/ISN and control vs HMC3 C.M; control vs HMC3 C.M/ISN); ^#^*P* < 0.05 (IL-6 vs IL-6/ISN and HMC3 C.M vs HMC3 C.M/ISN), *n* = 3; means ± SEM). HMC3 C.M: HMC3-conditional medium; ISN: isorhamnetin.

It has shown that IL-6 activates canonical JAK2/STAT3 and noncanonical TYK2/STAT1 pathways to upregulate neuronal apoptosis [21, 22]. Therefore, we explored the potential of isorhamnetin to demonstrate neuroprotection by modulating these pathways (Figure 5(a)). Treatment with IL-6 or HMC3-conditional medium enhanced the phosphorylation of TYK2 (IL-6 treatment, fold change: 2.03, *P* < 0.05; HMC3-conditioned medium treatment, fold change: 1.97, *P* < 0.01, compared with no treatment, Figures 5(b) and 5(c)) and STAT1 (IL-6 treatment, fold change: 3.05, *P* < 0.05; HMC3-conditioned medium treatment, fold change: 1.95, *P* < 0.01, compared with no treatment, Figures 5(b) and 5(d)), as well as increased the expression level of cleaved caspase 3 (IL-6 treatment, fold change: 1.81, *P* < 0.01; HMC3-conditioned medium treatment, fold change: 2.39, *P* < 0.001, compared with no treatment, Figures 5(b) and 5(e)). Isorhamnetin normalized the phosphorylation of TYK2 (fold change: 1.05, *P* < 0.05 compared with IL-6 only; fold change: 1.28, *P* < 0.05 compared with HMC3-conditioned medium only, Figures 5(b) and 5(c)), STAT1 (fold change: 0.93, *P* < 0.05 compared with IL-6 only; fold change: 1.37, *P* < 0.05 compared with HMC3-conditioned medium only, Figures 5(b) and 5(d)), and cleaved caspase 3 (fold change: 1.03, *P* < 0.05 compared with IL-6 only; fold change: 1.70, *P* < 0.05 compared with HMC3-conditioned medium only, Figures 5(b) and 5(e)). Furthermore, treatment with the TYK2 inhibitor consistently normalized the expression level of cleaved caspase 3 (HMC3-conditioned medium vs HMC3-conditioned medium/inhibitor: fold change: 1.81 vs 1.02, *P* < 0.001; IL-6 vs IL-6/inhibitor, fold change: 1.58 vs 1.17, *P* < 0.05, Supplementary Figure 4A-B). On the other hand, the phosphorylation of JAK2 and STAT3 was not affected by treatment with HMC3-conditioned medium, IL-6, or isorhamnetin (Figures 5(b), 5(f), and 5(g)). These results indicated that isorhamnetin provided neuroprotection against IL-6-mediated neurotoxicity by modulating the TYK2/STAT1 pathway.

### 3.5. Isorhamnetin Counteracted Apoptotic Activation and Preserved Neuronal Viability Even under TYK2 Overexpression

**Figure 6:**
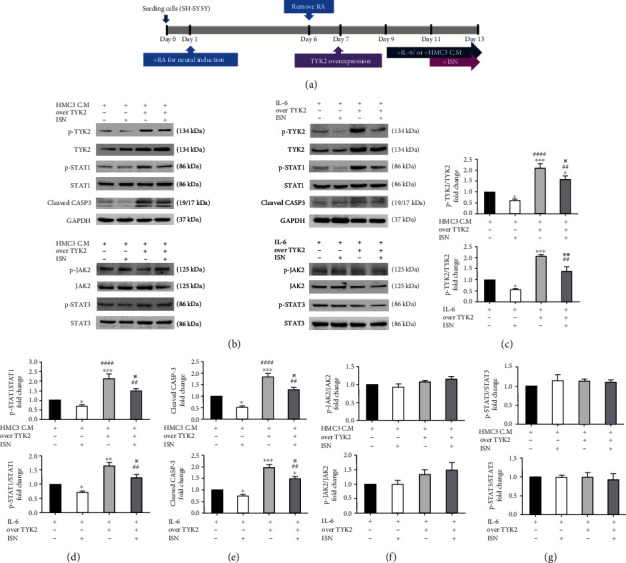
Isorhamnetin-induced neuroprotection attenuated the effect of TYK2 overexpression. (a) Experimental design scheme. (b–g) Immunoblots revealed TYK2/STAT1, JAK2/STAT3 signaling, and cleaved caspase 3 in HMC3-conditioned medium- or IL-6- (350 pg/mL) stimulated neurons treated with TYK2 overexpression and 10 *μ*M isorhamnetin. Isorhamnetin abrogated the activities of apoptosis and the TYK2/STAT1 pathway. Data were analyzed using one-way ANOVA with Bonferroni's post hoc test (^∗^*P* < 0.05, ^∗∗^*P* < 0.01, ^∗∗∗^*P* < 0.001 (HMC3 C.M vs HMC3 C.M/ISN; HMC3 C.M vs overTYK2/HMC3 C.M; HMC3 C.M vs overTYK2/HMC3 C.M/ISN and IL-6 vs IL-6/ISN; IL-6 vs overTYK2/IL-6; IL-6 vs overTYK2/IL-6/ISN); ^##^*P* < 0.01, ^####^*P* < 0.0001 (HMC3 C.M/ISN vs overTYK2/HMC3 C.M; HMC3 C.M/ISN vs overTYK2/HMC3 C.M/ISN and IL-6/ISN vs overTYK2/IL-6; IL-6/ISN vs overTYK2/IL-6/ISN); ^※^*P* < 0.05, ^※※^*P* < 0.01 (overTYK2/HMC3 C.M vs overTYK2/HMC3 C.M/ISN and overTYK2/IL-6 vs overTYK2/IL-6/ISN), *n* = 3; means ± SEM). HMC3 C.M: HMC3-conditioned medium; ISN: isorhamnetin; overTYK2: overexpressed TYK2.

**Figure 7:**
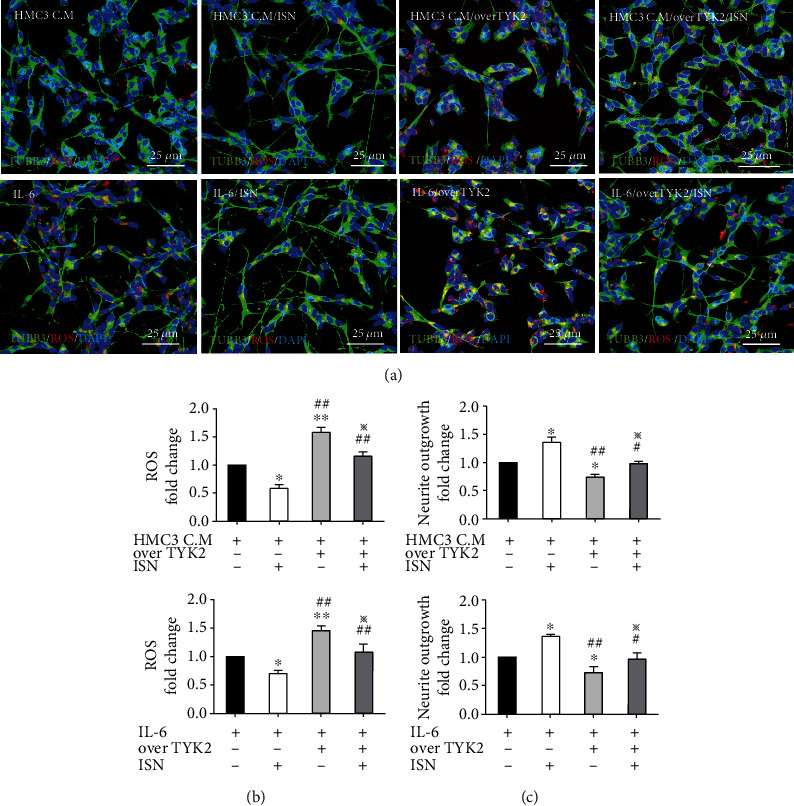
Isorhamnetin alleviated the impaired neurite growth and ROS overproduction induced by TYK2 overexpression. (a–c) Compared with TYK2 overexpression only, SH-SY5Y-derived neuronal cells cotreated with TYK2 overexpression and isorhamnetin (10 *μ*M) exhibited lower ROS intensity, as measured through fluorescent staining. Measurement of neurite outgrowth differed between cotreatment with TYK2 overexpression and isorhamnetin and TYK2 overexpression only, as examined using MetaMorph software. Scale bar, 25 *μ*m. Data were analyzed using one-way ANOVA with Bonferroni's post hoc test (^∗^*P* < 0.05, ^∗∗^*P* < 0.01 (HMC3 C.M vs HMC3 C.M/ISN; HMC3 C.M vs overTYK2/HMC3 C.M; HMC3 C.M vs overTYK2/HMC3 C.M/ISN and IL-6 vs IL-6/ISN; IL-6 vs overTYK2/IL-6; IL-6 vs overTYK2/IL-6/ISN); ^#^*P* < 0.05, ^##^*P* < 0.01 (HMC3 C.M/ISN vs overTYK2/HMC3 C.M; HMC3 C.M/ISN vs overTYK2/HMC3 C.M/ISN and IL-6/ISN vs overTYK2/IL-6; IL-6/ISN vs overTYK2/IL-6/ISN); ^※^*P* < 0.05 (overTYK2/HMC3 C.M vs overTYK2/HMC3 C.M/ISN and overTYK2/IL-6 vs overTYK2/IL-6/ISN), *n* = 3; means ± SEM). HMC3 C.M: HMC3-conditioned medium; ISN: isorhamnetin; overTYK2: overexpressed TYK2.

To confirm the role of TYK2 in the neuroprotective effect of isorhamnetin, we overexpressed TYK2 in the SH-ST5Y-derived neuronal cells under treatment with the HMC3-conditional medium, IL-6, and isorhamnetin (Figure 6(a)). Under treatment with either the HMC3-conditional medium or IL-6, TYK2 overexpression significantly increased the phosphorylation of STAT1 (HMC3-conditioned medium treatment, fold change: 2.13, *P* < 0.001; IL-6 treatment, fold change: 1.65, *P* < 0.01, Figures 6(b) and 6(d)), the expression level of cleaved caspase 3 (HMC3-conditional medium treatment, fold change: 1.83, *P* < 0.001; IL-6 treatment, fold change: 1.96, *P* < 0.001, Figures 6(b) and 6(e)), and the levels of ROS (HMC3-conditional medium treatment, fold change: 1.58, *P* < 0.01; IL-6 treatment, fold change: 1.45, *P* < 0.01, Figures 7(a) and 7(b)) as well as impaired neurite outgrowth (HMC3-conditional medium treatment, fold change: 0.74, *P* < 0.05; IL-6 treatment, fold change: 0.71, *P* < 0.05, Figures 7(a) and 7(c)). In addition, TYK2 overexpression counteracted the effects of isorhamnetin on the reduction of the phosphorylation of TYK2 (HMC3-conditioned medium/isorhamnetin vs overexpressed TYK2/HMC3-conditioned medium/isorhamnetin, fold change: 0.60 vs 1.57, *P* < 0.01; IL-6/isorhamnetin vs overexpressed TYK2/IL-6/isorhamnetin, fold change: 0.56 vs 1.38, *P* < 0.01, Figures 6(b) and 6(c)) and STAT1 (HMC3-conditioned medium/isorhamnetin vs overexpressed TYK2/HMC3-conditioned medium/isorhamnetin, fold change: 0.69 vs 1.49, *P* < 0.01; IL-6/isorhamnetin vs overexpressed TYK2/IL-6/isorhamnetin, fold change: 0.71 vs 1.23, *P* < 0.01, Figures 6(b) and 6(d)), the expression level of cleaved caspase 3 (HMC3-conditioned medium/isorhamnetin vs overexpressed TYK2/HMC3-conditioned medium/isorhamnetin, fold change: 0.52 vs 1.27, *P* < 0.01; IL-6/isorhamnetin vs overexpressed TYK2/IL-6/isorhamnetin, fold change: 0.74 vs 1.48, *P* < 0.01, Figures 6(b) and 6(e)), the level of ROS (HMC3-conditioned medium/isorhamnetin vs overexpressed TYK2/HMC3-conditioned medium/isorhamnetin, fold change: 0.59 vs 1.16, *P* < 0.01; IL-6/isorhamnetin vs overexpressed TYK2/IL-6/isorhamnetin, fold change: 0.71 vs 1.08, *P* < 0.01, Figures 7(a) and 7(b)), and the impairment of neurite outgrowth (HMC3-conditioned medium/isorhamnetin vs overexpressed TYK2/HMC3-conditioned medium/isorhamnetin, fold change: 1.40 vs 0.98, *P* < 0.05; IL-6/isorhamnetin vs overexpressed TYK2/IL-6/isorhamnetin, fold change: 1.36 vs 0.97, *P* < 0.05, Figures 7(a) and 7(c)). The phosphorylation of JAK2 and STAT3 was not affected by either TYK2 overexpression or isorhamnetin treatment (Figures 6(b), 6(f), and 6(g)). These results indicated that isorhamnetin exerted its neuroprotective effect by mediating TYK2 expression or phosphorylation.

## 4. Discussion

**Figure 8:**
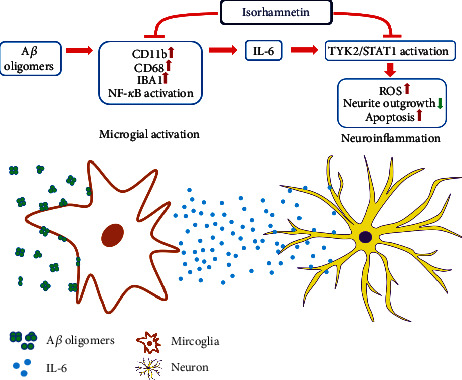
The anti-inflammatory and neuroprotective effects of isorhamnetin on Alzheimer's disease. A*β* oligomers activate microglia to secrete IL-6, which further upregulates the TYK2/STAT1 pathway to increase production of reactive oxygen species (ROS), impair neurite outgrowth, and upregulate apoptosis on neurons (neuroinflammation). Isorhamnetin attenuates microglial activation and IL-6 secretion and downregulates the TYK2/STAT1 pathway to reduce IL-6-mediated neuroinflammation.

Elucidating interactions between neuroinflammation and neurodegeneration can facilitate the development of new treatment strategies for AD. In this study, we established cell models to mimic neurodegenerative interactions among A*β*-activated microglia, inflammatory factors, and neurons. The results reveal that the A*β*-activated NF-*κ*B inflammatory signaling pathway upregulated the expression of the microglial activation markers CD11b, CD68, and IBA1 and increased the secretion of IL-6 from the HMC3 microglial cells. Both the HMC3-conditioned medium and IL-6 reduced cell viability, impaired neurite outgrowth, and increased ROS production and cleaved caspase 3 expression in the SH-SY5Y-derived neuronal cells. We observed that isorhamnetin attenuated the neuroinflammation and neurodegeneration induced by the HMC3-conditioned medium and IL-6. Furthermore, the results indicate that isorhamnetin suppressed neuroinflammation by deactivating the TYK2/STAT1 signaling pathway. These findings indicate the role of IL-6 in the nonautonomous interaction between microglia and neurons in AD. Furthermore, the findings demonstrate that the neuroprotective effect of isorhamnetin involves mediation of the noncanonical IL-6 signaling pathway, thus indicating the potential of isorhamnetin for use in AD treatment (Figure 8).

Our study demonstrates that IL-6 was the main constituent in the HMC3 conditional medium that regulated the neuroinflammatory interaction between microglia and neurons for neurodegeneration (Figures 2 and 4). Isorhamnetin suppressed the activation of the NF-*κ*B pathway, downregulated the expression of the microglial activation markers, and reduced the secretion of IL-6 from the A*β*-activated microglia (Figure 2). In lipopolysaccharide-induced BV2 mouse microglial cells, isorhamnetin reduced oxidative stress by reactivating TLR4 and the NF-*κ*B pathway [23]. Moreover, isorhamnetin was reported to attenuate oxidative stress and neurotoxicity by upregulating NRF2/HO-1 [24]. Similar to isorhamnetin, Gx-50, a natural compound derived from *Zanthoxylum bungeanum*, suppressed the activation of TLR4 and its downstream MyD88 and TRAF6 and reduced the expression levels of TNF-*α*, IL-1*β*, nitric oxide (NO), prostaglandin E2, inducible NO synthase, and cyclooxygenase-2 in A*β*-activated primary rat microglia and APP^+^ transgenic mice [7]. In addition, KHG26792, a novel azetidine derivative, attenuated the activation of the NF-*κ*B signaling pathway and reduced the expression levels of IL-6, IL-1*β*, TNF-*α*, NO, ROS, and NADHP oxidase in A*β*-treated rat primary microglia [25]. These compounds exert neuroprotective effects possibly by mediating neuroinflammation, and the neuroprotective potential of these compounds should be further explored in neuronal and animal models for AD.

The canonical downstream pathway of IL-6 in inflammatory cells and microglia involves the activation of JAK2 and STAT3. IL-6 upregulated the expression of STAT3 in rodent neurons [26, 27]. However, the downstream signaling of IL-6 in human neurons remains contentious. The phosphorylation of STAT3 was upregulated on SH-SY5Y cells exposed to a high IL-6 concentration (10 ng/mL) [27]. In this study, we observed that HMC3-conditioned medium and a low IL-6 concentration (350 pg/mL; this concentration was equal to that in the HMC3-conditioned medium) caused neurotoxicity without upregulating the JAK2/STAT3 signaling pathway in the SH-SY5Y-derived neuronal cells (Figure 5). However, the noncanonical TYK2/STAT1 phosphorylation was upregulated following treatment with a low IL-6 concentration. TYK2, a member of the JAK family, is associated with the intracellular domain of various cytokines that affect inflammatory responses [28]. In addition, the activation of the TYK2/STAT signaling pathway is associated with inflammatory responses, apoptosis, and ROS production [21, 22, 29]. Knockout of TYK2 mitigated neuronal death in APP/PS1 transgenic mice [23]. Our results indicate that isorhamnetin downregulated TYK2/STAT1 phosphorylation to attenuate the neurotoxic effects of IL-6, whereas TYK2 overexpression attenuated the neuroprotective effect of isorhamnetin (Figures 6 and 7). These results highlight the role of TYK2/STAT1 signaling in AD pathogenesis and isorhamnetin in protection against neuroinflammation.

Isorhamnetin, the main constituent of flavonoids, possesses antioxidative properties [30, 31]. In addition to protection against neuroinflammation, isorhamnetin exerts antioxidative and neurotropic effects. Isorhamnetin potentiated the effect of the nerve growth factor on the neurite outgrowth of PC12 cells [32]. In mice treated with scopolamine, isorhamnetin reduced oxidative stress and suppressed cholinergic signaling pathways, reduced cholinesterase activity [33], and improved impairment in spatial and nonspatial learning and memory [34]. Furthermore, treatment with isorhamnetin in stroke mice improved the integrity of the blood-brain barrier and reduced the levels of IL-1*β*, IL-6, and TNF-*α* in the ischemic cortex [35]. Together with our findings, these results suggest the pleiotropic effects of isorhamnetin for neuroprotection. Isorhamnetin can effectively penetrate the blood-brain barrier [36] and thus can be used for treating neurological diseases. Furthermore, our results reveal that isorhamnetin exhibite neuroprotection in the SH-SY5Y-derived neuronal cells following IL-6 treatment, suggesting that isorhamnetin can reduce neuroinflammation-mediated neurodegeneration.

In conclusion, our study indicates the pivotal role of IL-6 in mediating nonautonomous interactions between microglia and neurons in AD and that the TYK2/STAT1 signaling pathway can be a target for treating neuroinflammation in AD. In addition, our results demonstrate that isorhamnetin alleviates neuroinflammation by deactivating the TYK2/STAT1 signaling pathway. These findings provide new insights into neuroinflammatory pathogenesis as well as a novel therapeutic target for AD. Additional animal studies should be conducted to validate our findings.

## Data Availability

The datasets generated during the current study are available from the corresponding author upon reasonable request.

## Supplementary Materials

Supplementary Materials Supplementary Figure 1: time-response experiments of A*β* oligomers and isorhamnetin in HMC3 cells. (A) Treatment with A*β* oligomers (200 nM) for 24 or 48 hours increased IL-6 release, whereas the secretions of IL-1*β* and TNF-*α* were not affected by A*β* oligomers. (B) Experimental flowchart. HMC3 cells activated by A*β* were treated with isorhamnetin (10 *μ*M) for 24 or 48 hours. (C) In A*β*-activated HMC3 cells, treatment with isorhamnetin for 24 hours did not reduce the secretion of IL-6, while 48-hour treatment reduced the IL-6 secretion to baseline. Data were analyzed using one-way ANOVA with Bonferroni's post hoc test (^∗^*P* < 0.05, ^∗∗^*P* < 0.01 (control vs A*β*); ^##^*P* < 0.05 (A*β* vs A*β*/ISN), *n* = 3; means ± SEM). A*β*: A*β* oligomers; ISN: isorhamnetin. Supplementary Figure 2: NF-*κ*B inhibitor suppressed the A*β*-induced activation of HMC3 cells. (A) Western blot in HMC3 cells treated with A*β* oligomers (200 nM) or EVP4593 (NF-*κ*B inhibitor, 1 *μ*M). Treatment with EVP4593 for 48 hours reduced phosphorylation of NF-*κ*B (B), the expression of CD11b (C), CD68 (D), IBA1(E), and IL-6 secretion (F). Data were analyzed using one-way ANOVA with Bonferroni's post hoc test or Student's *t*-test (^∗^*P* < 0.05, ^∗∗^*P* < 0.01, and ^∗∗∗^*P* < 0.001 (control vs A*β*); ^#^*P* < 0.05, ^##^*P* < 0.01, and ^###^*P* < 0.001 (A*β* vs A*β*/EVP4593), *n* = 3; means ± SEM). A*β*: A*β* oligomers; EVP4593: NF-*κ*B inhibitor. Supplementary Figure 3: IL-6 IgG neutralized the effect of HMC3 activated-conditional medium on SH-SY5Y-derived neurons. (A-B) IL-6 IgG (5 ng/mL for 48 hours) reduced the expression cleaved caspase 3 in SH-SY5Y-derived neurons treated with HMC3-conditioned medium. (C-D) IL-6 IgG reduced ROS production in SH-SY5Y-derived neurons treated with HMC3-conditioned medium. (E) The impaired neurite outgrowth by HMC3-conditioned medium was rescued by IL-6-neutralizing antibody. Images were measured using MetaMorph software. Scale bar, 25 *μ*m. Data were analyzed using one-way ANOVA with Bonferroni's post hoc test (^∗^*P* < 0.05, ^∗∗∗^*P* < 0.001 (control vs HMC3 C.M; control vs HMC3 C.M/IL-6 IgG); ^#^*P* < 0.05 (HMC3 C.M vs HMC3 C.M/IL-6 IgG), *n* = 3; means ± SEM). HMC3 C.M: HMC3-conditioned medium; IL-6 IgG: IL-6 neutralized antibody. Supplementary Figure 4: TYK2 inhibitor suppressed IL-6 or HMC3-conditioned medium induced apoptosis in SH-SY5Y-derived neurons. (A-B) In SH-SY5Y-derived neurons, the upregulation of cleaved caspase 3 by IL-6 (350 pg/mL) or HMC3-conditioned medium was counteracted by treatment with the TYK2 inhibitor (1 *μ*M) for 48 hours. Data were analyzed using one-way ANOVA with Bonferroni's post hoc test (^∗∗^*P* < 0.01, ^∗∗∗^*P* < 0.001 (control vs IL-6; control vs HMC3 C.M); ^#^*P* < 0.05 (IL-6 vs IL-6/TYK2 inhibitor); ^※※※^*P* < 0.001 (HMC3 C.M vs HMC3 C.M/TYK2 inhibitor), *n* = 3; means ± SEM). HMC3 C.M: HMC3 activated-conditioned medium.
