# Supplementary material for: Isorhamnetin Attenuated the Release of Interleukin-6 from β-Amyloid-Activated Microglia and Mitigated Interleukin-6-Mediated Neurotoxicity
Source: Oxid Med Cell Longev. 2022 Sep 15;2022:3652402. doi: 10.1155/2022/3652402 (PMC9499806; doi:10.1155/2022/3652402)
Supplement: Supplementary Materials — Supplementary Figure 1: time-response experiments of Aβ oligomers and isorhamnetin in HMC3 cells. (A) Treatment with Aβ oligomers (200 nM) for 24 or 48 hours increased IL-6 release, whereas the secretions of IL-1β and TNF-α were not affected by Aβ oligomers. (B) Experimental flowchart. HMC3 cells activated by Aβ were treated with isorhamnetin (10 μM) for 24 or 48 hours. (C) In Aβ-activated HMC3 cells, treatment with isorhamnetin for 24 hours did not reduce the secretion of IL-6, while 48-hour treatment reduced the IL-6 secretion to baseline. Data were analyzed using one-way ANOVA with Bonferroni's post hoc test (∗P < 0.05, ∗∗P < 0.01 (control vs Aβ); ##P < 0.05 (Aβ vs Aβ/ISN), n = 3; means ± SEM). Aβ: Aβ oligomers; ISN: isorhamnetin. Supplementary Figure 2: NF-κB inhibitor suppressed the Aβ-induced activation of HMC3 cells. (A) Western blot in HMC3 cells treated with Aβ oligomers (200 nM) or EVP4593 (NF-κB inhibitor, 1 μM). Treatment with EVP4593 for 48 hours reduced phosphorylation of NF-κB (B), the expression of CD11b (C), CD68 (D), IBA1(E), and IL-6 secretion (F). Data were analyzed using one-way ANOVA with Bonferroni's post hoc test or Student's t-test (∗P < 0.05, ∗∗P < 0.01, and ∗∗∗P < 0.001 (control vs Aβ); #P < 0.05, ##P < 0.01, and ###P < 0.001 (Aβ vs Aβ/EVP4593), n = 3; means ± SEM). Aβ: Aβ oligomers; EVP4593: NF-κB inhibitor. Supplementary Figure 3: IL-6 IgG neutralized the effect of HMC3 activated-conditional medium on SH-SY5Y-derived neurons. (A-B) IL-6 IgG (5 ng/mL for 48 hours) reduced the expression cleaved caspase 3 in SH-SY5Y-derived neurons treated with HMC3-conditioned medium. (C-D) IL-6 IgG reduced ROS production in SH-SY5Y-derived neurons treated with HMC3-conditioned medium. (E) The impaired neurite outgrowth by HMC3-conditioned medium was rescued by IL-6-neutralizing antibody. Images were measured using MetaMorph software. Scale bar, 25 μm. Data were analyzed using one-way ANOVA with Bonferroni's post hoc test (∗P < 0.05, ∗∗∗P < 0.001 (control [file 3652402.f1.zip › Supplementary information (1).pdf]

Supplementary information for:

Isorhamnetin attenuated the release of interleukin-6 from β-amyloid-activated microglia and mitigated interleukin-6-mediated neurotoxicity

Pei-Cih Wei[1], Guey-Jen Lee-Chen[2], Chiung-Mei Chen[1], Ying Chen[1], Yen-Shi Lo[1], Yi-Jing Chen[1], *Kuo-Hsuan Chang[1]

[1]Department of Neurology, Chang Gung Memorial Hospital-Linkou Medical Center, Chang Gung University School of Medicine, Taoyuan, Taiwan

[2]Department of Life Science, National Taiwan Normal University, Taipei, Taiwan

Correspondence:

*Kuo-Hsuan Chang, MD., Ph.D.

Phone: 886 (0) 33281200 ext. 5092

Fax: 886 (0) 33288849

E-mail: gophy5128@cgmh.org.tw

Supplementary figure legends

Supplementary Fig. 1. Time-response experiments of Aβ oligomers and isorhamnetin in HMC3 cells. (A) Treatment with Aβ oligomers (200 nM) for 24 or 48 hours increased IL-6 release, whereas the secretions of IL-1β and TNF-α were not affected by Aβ oligomers. (B) Experimental flowchart. HMC3 cells activated by Aβ were treated with isorhamnetin (10 μM) for 24 or 48 hours. (C) In Aβ-activated HMC3 cells, treatment with isorhamnetin for 24 hours did not reduce the secretion of IL-6, while 48 hours treatment reduced the IL-6 secretion to baseline. Data were analyzed using one-way ANOVA with Bonferroni's post hoc test ($^{*}P < 0.05$, $^{**}P < 0.01$ [control vs. Aβ]; $^{\#\#}P < 0.05$ [Aβ vs. Aβ /ISN], n = 3; means ± SEM). Aβ: Aβ oligomers; ISN: isorhamnetin.

Supplementary Fig. 2. NF-κB inhibitor suppressed the Aβ-induced activation of HMC3 cells. (A) Western blot in HMC3 cells treated with Aβ oligomers (200 nM) or EVP4593 (NF-κB inhibitor, 1 μM). Treatment with EVP4593 for 48 hours reduced phosphorylation of NF-κB (B), the expression of CD11b (C), CD68 (D), IBA1(E), and IL-6 secretion (F). Data were analyzed using one-way ANOVA with Bonferroni's post hoc test or Student's t test ($^{*}P < 0.05$, $^{**}P < 0.01$, $^{***}P < 0.001$ [control vs. Aβ]; $^{\#}P < 0.05$, $^{\#\#}P < 0.01$, $^{\#\#\#}P < 0.001$ [Aβ vs. Aβ/EVP4593], n = 3; means ± SEM). Aβ: Aβ oligomers; EVP4593: NF-κB inhibitor.

Supplementary Fig. 3. IL-6 IgG neutralized the effect of HMC3 activated-conditional medium in SH-SY5Y-derived neurons. (A-B) IL-6 IgG (5 ng/ml for 48 hours) reduced the expression cleaved caspase 3 in SH-SY5Y-derived neurons treated with HMC3-conditioned medium. (C-D) IL-6 IgG reduced ROS production in SH-SY5Y-derived neurons treated with HMC3-conditioned medium. (E) The impaired

neurite outgrowth by HMC3 conditioned medium was rescued by IL-6 neutralizing antibody. Images were measured using MetaMorph software. Scale bar, 25μm. Data were analyzed using one-way ANOVA with Bonferroni's post hoc test (*$P < 0.05$, ***$P < 0.001$ [control vs. HMC3 C.M; control vs. HMC3 C.M/IL-6 IgG]; $^{\#}P < 0.05$ [HMC3 C.M vs. HMC3 C.M/IL-6 IgG], n = 3; means ± SEM). HMC3 C.M: HMC3-conditioned medium; IL-6 IgG: IL-6 neutralized antibody.

Supplementary Fig. 4. TYK2 inhibitor suppressed IL-6 or HMC3 conditioned medium induced apoptosis in SH-SY5Y-derived neurons. (A-B) In SH-SY5Y-derived neurons, the upregulation of cleaved caspase 3 by IL-6 (350 pg/mL) or HMC3 conditioned medium was counteracted by treatment with TYK2 inhibitor (1 μM) for 48 hours. Data were analyzed using one-way ANOVA with Bonferroni's post hoc test (**$P < 0.01$, ***$P < 0.001$ [control vs. IL-6; control vs. HMC3 C.M]; $^{\#}P < 0.05$ [IL-6 vs. IL-6/TYK2 inhibitor]; ***$P < 0.001$ [HMC3 C.M vs. HMC3 C.M/TYK2 inhibitor], n = 3; means ± SEM). HMC3 C.M: HMC3 activated-conditioned medium.
