# Supplementary figures and images for: Isorhamnetin Attenuated the Release of Interleukin-6 from β-Amyloid-Activated Microglia and Mitigated Interleukin-6-Mediated Neurotoxicity
Source: Oxid Med Cell Longev. 2022 Sep 15;2022:3652402. doi: 10.1155/2022/3652402 (PMC9499806; doi:10.1155/2022/3652402)

A.

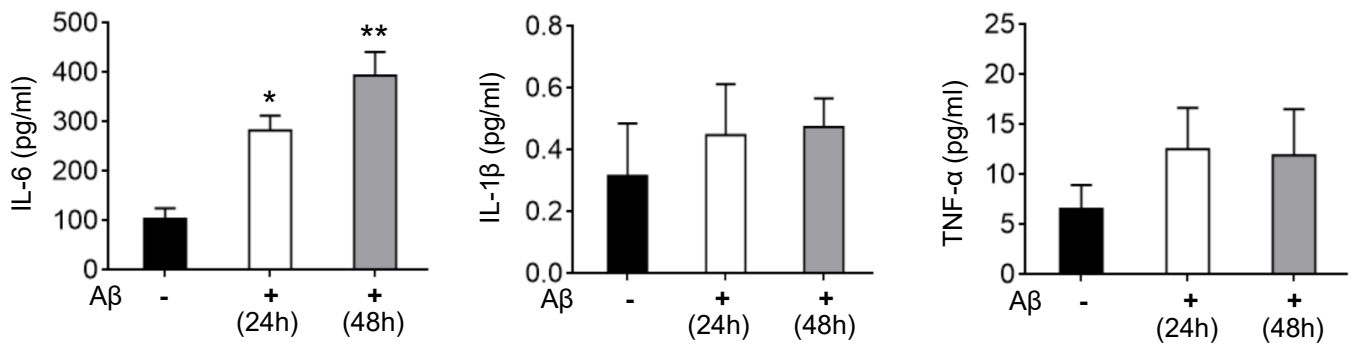

B.

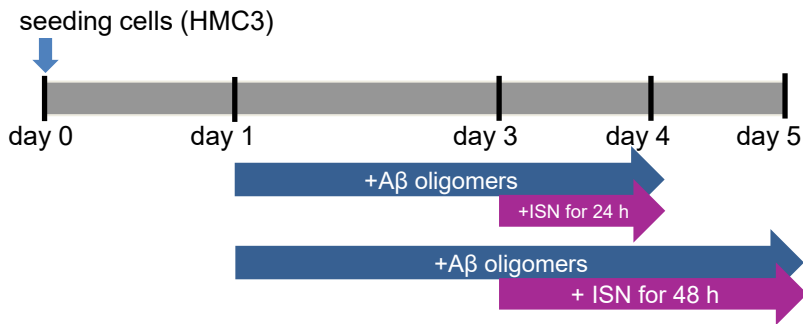

C.

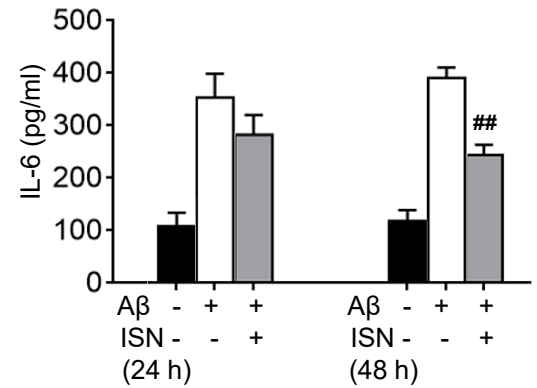

Supplement: Supplementary Materials — Supplementary Figure 1: time-response experiments of Aβ oligomers and isorhamnetin in HMC3 cells. (A) Treatment with Aβ oligomers (200 nM) for 24 or 48 hours increased IL-6 release, whereas the secretions of IL-1β and TNF-α were not affected by Aβ oligomers. (B) Experimental flowchart. HMC3 cells activated by Aβ were treated with isorhamnetin (10 μM) for 24 or 48 hours. (C) In Aβ-activated HMC3 cells, treatment with isorhamnetin for 24 hours did not reduce the secretion of IL-6, while 48-hour treatment reduced the IL-6 secretion to baseline. Data were analyzed using one-way ANOVA with Bonferroni's post hoc test (∗P < 0.05, ∗∗P < 0.01 (control vs Aβ); ##P < 0.05 (Aβ vs Aβ/ISN), n = 3; means ± SEM). Aβ: Aβ oligomers; ISN: isorhamnetin. Supplementary Figure 2: NF-κB inhibitor suppressed the Aβ-induced activation of HMC3 cells. (A) Western blot in HMC3 cells treated with Aβ oligomers (200 nM) or EVP4593 (NF-κB inhibitor, 1 μM). Treatment with EVP4593 for 48 hours reduced phosphorylation of NF-κB (B), the expression of CD11b (C), CD68 (D), IBA1(E), and IL-6 secretion (F). Data were analyzed using one-way ANOVA with Bonferroni's post hoc test or Student's t-test (∗P < 0.05, ∗∗P < 0.01, and ∗∗∗P < 0.001 (control vs Aβ); #P < 0.05, ##P < 0.01, and ###P < 0.001 (Aβ vs Aβ/EVP4593), n = 3; means ± SEM). Aβ: Aβ oligomers; EVP4593: NF-κB inhibitor. Supplementary Figure 3: IL-6 IgG neutralized the effect of HMC3 activated-conditional medium on SH-SY5Y-derived neurons. (A-B) IL-6 IgG (5 ng/mL for 48 hours) reduced the expression cleaved caspase 3 in SH-SY5Y-derived neurons treated with HMC3-conditioned medium. (C-D) IL-6 IgG reduced ROS production in SH-SY5Y-derived neurons treated with HMC3-conditioned medium. (E) The impaired neurite outgrowth by HMC3-conditioned medium was rescued by IL-6-neutralizing antibody. Images were measured using MetaMorph software. Scale bar, 25 μm. Data were analyzed using one-way ANOVA with Bonferroni's post hoc test (∗P < 0.05, ∗∗∗P < 0.001 (control [file 3652402.f1.zip › Fig. S1 (1).pdf]

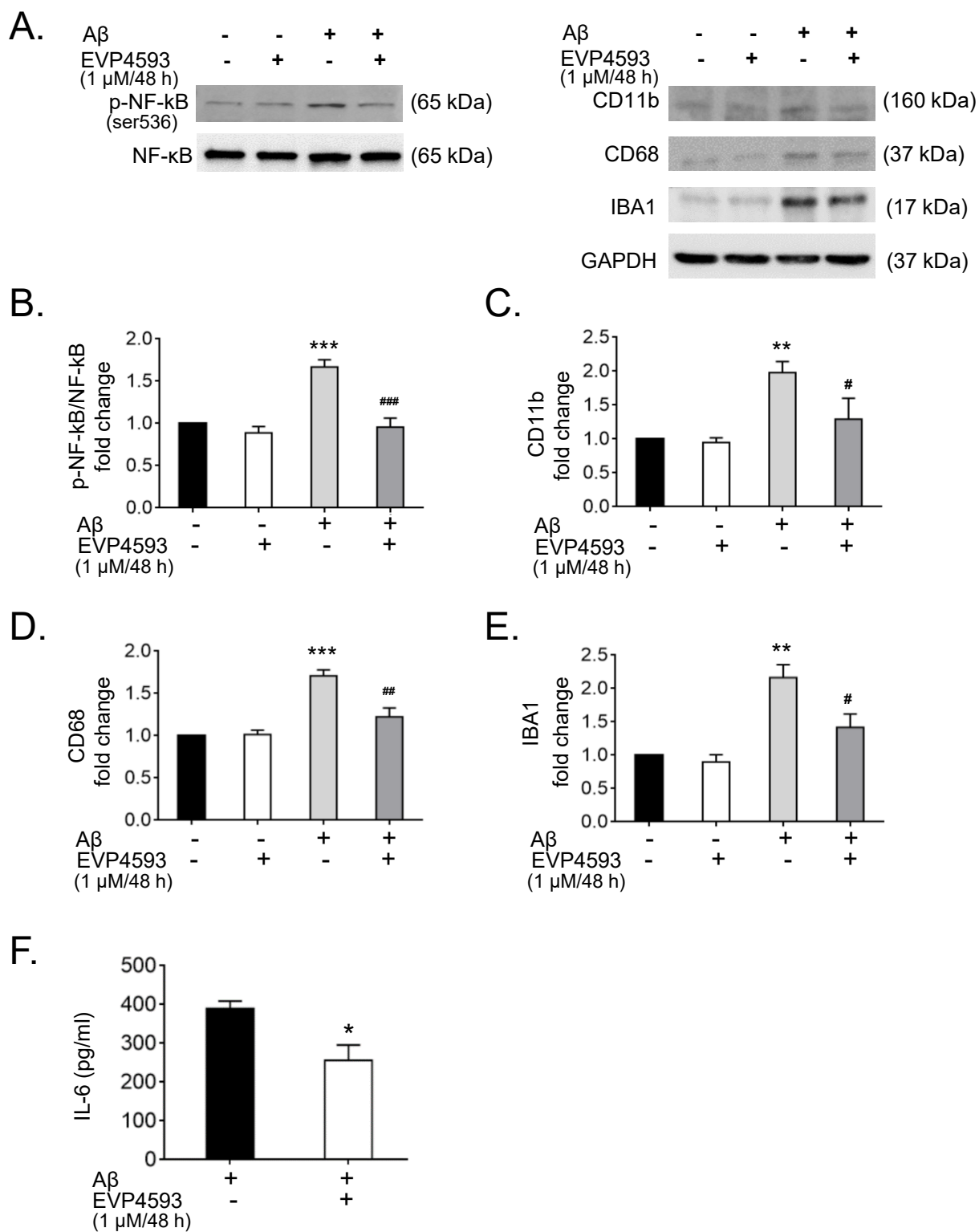

Supplement: Supplementary Materials — Supplementary Figure 1: time-response experiments of Aβ oligomers and isorhamnetin in HMC3 cells. (A) Treatment with Aβ oligomers (200 nM) for 24 or 48 hours increased IL-6 release, whereas the secretions of IL-1β and TNF-α were not affected by Aβ oligomers. (B) Experimental flowchart. HMC3 cells activated by Aβ were treated with isorhamnetin (10 μM) for 24 or 48 hours. (C) In Aβ-activated HMC3 cells, treatment with isorhamnetin for 24 hours did not reduce the secretion of IL-6, while 48-hour treatment reduced the IL-6 secretion to baseline. Data were analyzed using one-way ANOVA with Bonferroni's post hoc test (∗P < 0.05, ∗∗P < 0.01 (control vs Aβ); ##P < 0.05 (Aβ vs Aβ/ISN), n = 3; means ± SEM). Aβ: Aβ oligomers; ISN: isorhamnetin. Supplementary Figure 2: NF-κB inhibitor suppressed the Aβ-induced activation of HMC3 cells. (A) Western blot in HMC3 cells treated with Aβ oligomers (200 nM) or EVP4593 (NF-κB inhibitor, 1 μM). Treatment with EVP4593 for 48 hours reduced phosphorylation of NF-κB (B), the expression of CD11b (C), CD68 (D), IBA1(E), and IL-6 secretion (F). Data were analyzed using one-way ANOVA with Bonferroni's post hoc test or Student's t-test (∗P < 0.05, ∗∗P < 0.01, and ∗∗∗P < 0.001 (control vs Aβ); #P < 0.05, ##P < 0.01, and ###P < 0.001 (Aβ vs Aβ/EVP4593), n = 3; means ± SEM). Aβ: Aβ oligomers; EVP4593: NF-κB inhibitor. Supplementary Figure 3: IL-6 IgG neutralized the effect of HMC3 activated-conditional medium on SH-SY5Y-derived neurons. (A-B) IL-6 IgG (5 ng/mL for 48 hours) reduced the expression cleaved caspase 3 in SH-SY5Y-derived neurons treated with HMC3-conditioned medium. (C-D) IL-6 IgG reduced ROS production in SH-SY5Y-derived neurons treated with HMC3-conditioned medium. (E) The impaired neurite outgrowth by HMC3-conditioned medium was rescued by IL-6-neutralizing antibody. Images were measured using MetaMorph software. Scale bar, 25 μm. Data were analyzed using one-way ANOVA with Bonferroni's post hoc test (∗P < 0.05, ∗∗∗P < 0.001 (control [file 3652402.f1.zip › Fig. S2 (1).pdf]

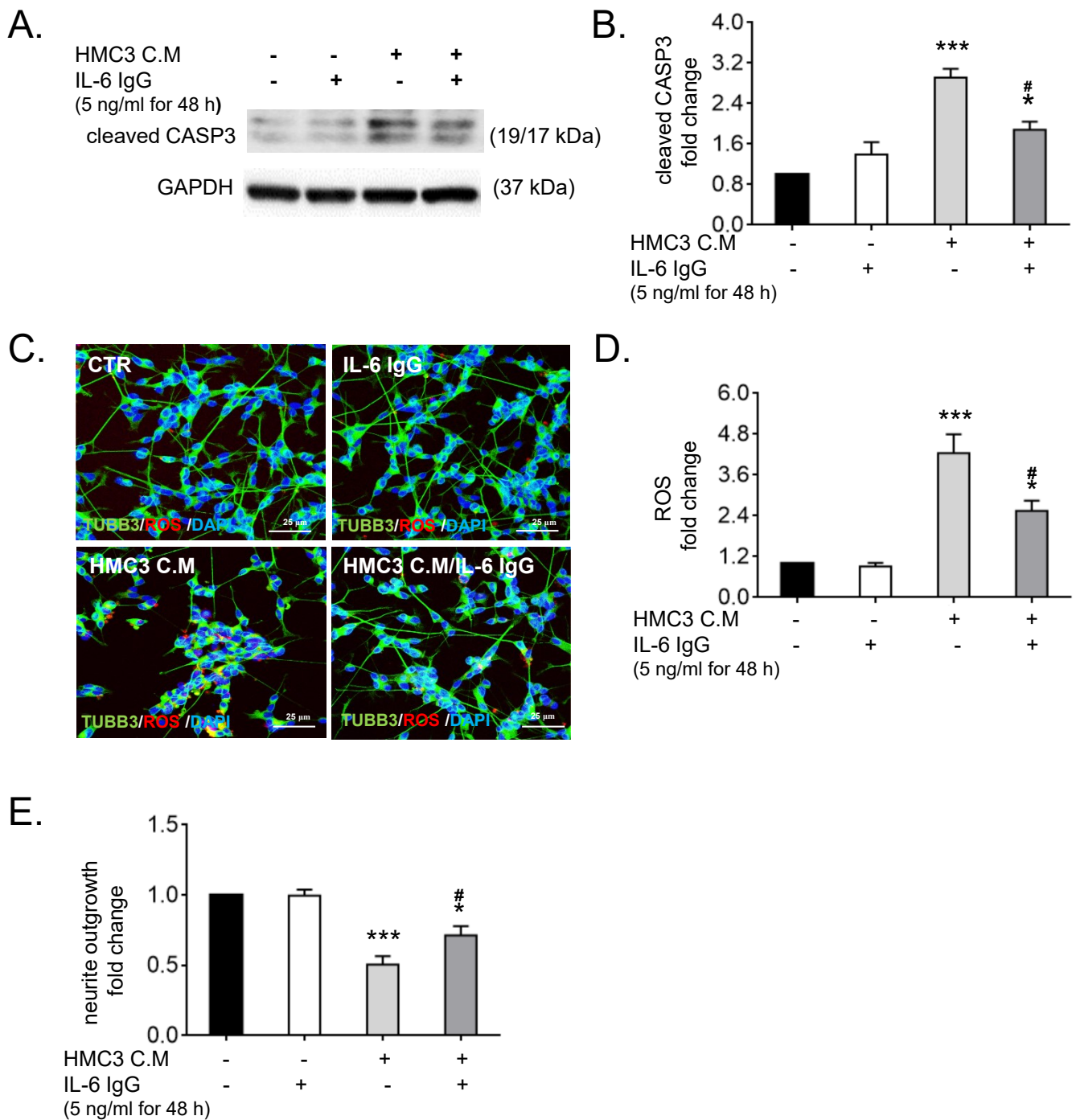

Supplement: Supplementary Materials — Supplementary Figure 1: time-response experiments of Aβ oligomers and isorhamnetin in HMC3 cells. (A) Treatment with Aβ oligomers (200 nM) for 24 or 48 hours increased IL-6 release, whereas the secretions of IL-1β and TNF-α were not affected by Aβ oligomers. (B) Experimental flowchart. HMC3 cells activated by Aβ were treated with isorhamnetin (10 μM) for 24 or 48 hours. (C) In Aβ-activated HMC3 cells, treatment with isorhamnetin for 24 hours did not reduce the secretion of IL-6, while 48-hour treatment reduced the IL-6 secretion to baseline. Data were analyzed using one-way ANOVA with Bonferroni's post hoc test (∗P < 0.05, ∗∗P < 0.01 (control vs Aβ); ##P < 0.05 (Aβ vs Aβ/ISN), n = 3; means ± SEM). Aβ: Aβ oligomers; ISN: isorhamnetin. Supplementary Figure 2: NF-κB inhibitor suppressed the Aβ-induced activation of HMC3 cells. (A) Western blot in HMC3 cells treated with Aβ oligomers (200 nM) or EVP4593 (NF-κB inhibitor, 1 μM). Treatment with EVP4593 for 48 hours reduced phosphorylation of NF-κB (B), the expression of CD11b (C), CD68 (D), IBA1(E), and IL-6 secretion (F). Data were analyzed using one-way ANOVA with Bonferroni's post hoc test or Student's t-test (∗P < 0.05, ∗∗P < 0.01, and ∗∗∗P < 0.001 (control vs Aβ); #P < 0.05, ##P < 0.01, and ###P < 0.001 (Aβ vs Aβ/EVP4593), n = 3; means ± SEM). Aβ: Aβ oligomers; EVP4593: NF-κB inhibitor. Supplementary Figure 3: IL-6 IgG neutralized the effect of HMC3 activated-conditional medium on SH-SY5Y-derived neurons. (A-B) IL-6 IgG (5 ng/mL for 48 hours) reduced the expression cleaved caspase 3 in SH-SY5Y-derived neurons treated with HMC3-conditioned medium. (C-D) IL-6 IgG reduced ROS production in SH-SY5Y-derived neurons treated with HMC3-conditioned medium. (E) The impaired neurite outgrowth by HMC3-conditioned medium was rescued by IL-6-neutralizing antibody. Images were measured using MetaMorph software. Scale bar, 25 μm. Data were analyzed using one-way ANOVA with Bonferroni's post hoc test (∗P < 0.05, ∗∗∗P < 0.001 (control [file 3652402.f1.zip › Fig. S3 (1).pdf]

A.

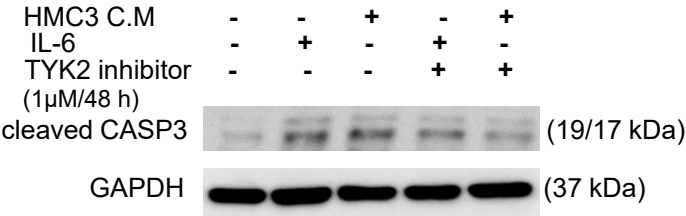

B.

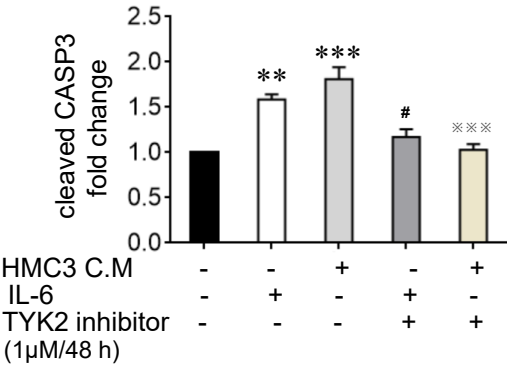

Supplement: Supplementary Materials — Supplementary Figure 1: time-response experiments of Aβ oligomers and isorhamnetin in HMC3 cells. (A) Treatment with Aβ oligomers (200 nM) for 24 or 48 hours increased IL-6 release, whereas the secretions of IL-1β and TNF-α were not affected by Aβ oligomers. (B) Experimental flowchart. HMC3 cells activated by Aβ were treated with isorhamnetin (10 μM) for 24 or 48 hours. (C) In Aβ-activated HMC3 cells, treatment with isorhamnetin for 24 hours did not reduce the secretion of IL-6, while 48-hour treatment reduced the IL-6 secretion to baseline. Data were analyzed using one-way ANOVA with Bonferroni's post hoc test (∗P < 0.05, ∗∗P < 0.01 (control vs Aβ); ##P < 0.05 (Aβ vs Aβ/ISN), n = 3; means ± SEM). Aβ: Aβ oligomers; ISN: isorhamnetin. Supplementary Figure 2: NF-κB inhibitor suppressed the Aβ-induced activation of HMC3 cells. (A) Western blot in HMC3 cells treated with Aβ oligomers (200 nM) or EVP4593 (NF-κB inhibitor, 1 μM). Treatment with EVP4593 for 48 hours reduced phosphorylation of NF-κB (B), the expression of CD11b (C), CD68 (D), IBA1(E), and IL-6 secretion (F). Data were analyzed using one-way ANOVA with Bonferroni's post hoc test or Student's t-test (∗P < 0.05, ∗∗P < 0.01, and ∗∗∗P < 0.001 (control vs Aβ); #P < 0.05, ##P < 0.01, and ###P < 0.001 (Aβ vs Aβ/EVP4593), n = 3; means ± SEM). Aβ: Aβ oligomers; EVP4593: NF-κB inhibitor. Supplementary Figure 3: IL-6 IgG neutralized the effect of HMC3 activated-conditional medium on SH-SY5Y-derived neurons. (A-B) IL-6 IgG (5 ng/mL for 48 hours) reduced the expression cleaved caspase 3 in SH-SY5Y-derived neurons treated with HMC3-conditioned medium. (C-D) IL-6 IgG reduced ROS production in SH-SY5Y-derived neurons treated with HMC3-conditioned medium. (E) The impaired neurite outgrowth by HMC3-conditioned medium was rescued by IL-6-neutralizing antibody. Images were measured using MetaMorph software. Scale bar, 25 μm. Data were analyzed using one-way ANOVA with Bonferroni's post hoc test (∗P < 0.05, ∗∗∗P < 0.001 (control [file 3652402.f1.zip › supplemental fig. 4.pdf]
